
JOURNAL INFORMATION
==============================
NLM Title Abbreviation: ScientificWorldJournal
Journal ID: ScientificWorldJournal
NLM Journal ID: 101131163
Journal ID: TSWJ

The Scientific World Journal

ISSN: 2356-6140
EISSN: 1537-744X
Publisher: Wiley

ARTICLE INFORMATION
==============================
PMCID: PMC6083997
PMID: 30147466
DOI: 10.1100/tsw.2001.154
Article ID: 467537
Article version: 1
Subjects: Short Report

Apoptosis Gene Hunting Using Retroviral Expression Cloning

Anderson Claire L. *
Williams Gwyn T.
School of Life Sciences, Keele University, Keele, Staffs, ST5 5BG, England
*Claire L. Anderson: c.l.anderson@biol.keele.ac.uk
Publication date: 2001
Electronic publication date: 2001 Oct 23
Volume: 1
First page: 33
Last page: 33
Copyright: Copyright © 2001 Claire L. Anderson and Gwyn T. Williams.
Copyright year: 2001
License: This is an open access article distributed under the Creative Commons Attribution License, which permits unrestricted use, distribution, and reproduction in any medium, provided the original work is properly cited.
License URL: https://creativecommons.org/licenses/by/3.0/

==============================
Miami *Nature Biotechnology* Short Reports
*TheScientificWorld* (2001) 1(S3), 33SR
ISSN 1532-2246; DOI 10.1100/tsw.2001.154

### APOPTOSIS GENE HUNTING USING RETROVIRAL EXPRESSION CLONING

Claire L. Anderson* and Gwyn T. Williams

School of Life Sciences, Keele University, Keele, Staffs, ST5 5BG, England
* c.l.anderson@biol.keele.ac.uk

**INTRODUCTION.** cDNA expression cloning is a powerful technique for the isolation of genes, which confer selectable phenotypes on specific cell types. This strategy has been used successfully to identify potential oncogenes (1), genes capable of suppressing cytokine signal transduction (2) and genes encoding cell surface antigens (3). We have utilised a functional expression cloning strategy in conjunction with a retroviral vector, pRUF*neo*, to search for important regulatory genes involved in apoptosis. This approach has the advantage that it assumes no prior involvement in apoptosis or makes any assumptions concerning nucleotide sequence or protein-protein interactions.

**METHOD.** BAF-3 cells, a murine interleukin (IL)-3-dependent cell line of pro-B lymphocytes, were infected with the retroviral vector, pRUF*neo*, into which a cDNA library from the factor dependent cell line FDC-P1 had been inserted (4). IL-3 deprivation of retrovirally infected BAF-3 cells was used to identify cDNAs that conferred resistance to IL-3 withdrawal-induced apoptosis, by plating in soft agar in the presence of IL-3 and geneticin. Genomic DNA from IL-3 resistant clones was isolated and amplified by PCR using pRUF*neo* vector primers to recover cDNA inserts, which were subsequently identified by sequencing.

**RESULTS**. Several IL-3 resistant clones were isolated (Fig. 1) and cDNA inserts were recovered by PCR (Fig. 2). Sequence analysis of these inserts revealed a 3' untranslated portion of the vacuolar ATPase subunit E gene, ribosomal protein L17 and saposin, in addition to several putatively novel sequences. Functional analysis of the degree of apoptosis resistance conferred by these cDNA inserts will be presented.

A. Parental BAF-3 cells
(i) No G418
(ii) 1 mg/ml G418
B. Retrovirally infected BAF-3 cells
(i) No G418
(ii) 1 mg/ml G418

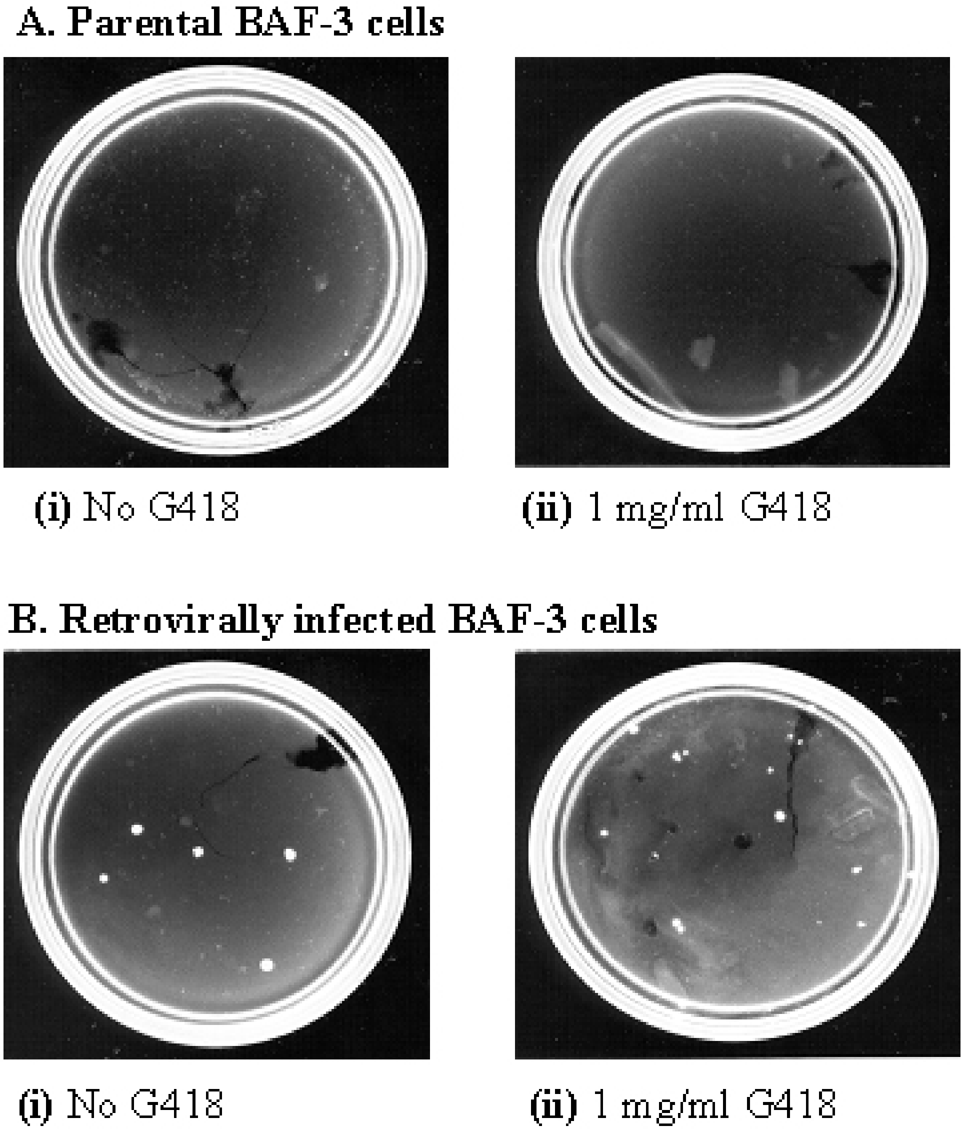

Figure 1

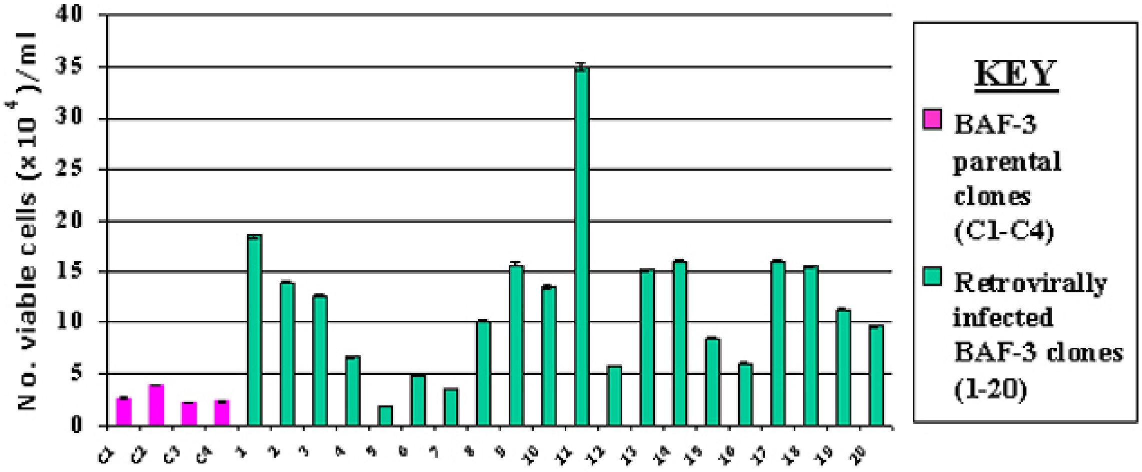

Figure 2

**DISCUSSION**. We have shown that cDNA expression cloning utilising a retroviral vector system can be successfully used to isolate potentially novel apoptosis controlling genes, as well as known genes whose role in IL-3 withdrawal-induced apoptosis has not previously been demonstrated.

**ACKNOWLEDGEMENTS**. This work was supported by a Leukaemia Research Fund grant (to C.L.A.). The cDNA library was kindly donated by Thomas Gonda and colleagues, Hanson Institute, Adelaide, Australia. We thank our colleagues at the Walter and Elisa Hall Institute, Melbourne, at King's College Hospital Medical School, London and at the School of Life Sciences, Keele University for valuable help and advice.

**REFERENCES.**

1. Whitehead, I., Kirk, H., and Kay, R. (1995) Mol. Cell. Biol. 15, 704-710
2. Starr, R., Willson, T., Viney, E., et al. (1997) Nature 387, 917-921
3. Zannettino, A., Rayner, J., Ashman, L., et al. (1996) J. Immunol. 156, 611-620
4. Raynor, J. and Gonda, T. (1994) Mol. Cell. Biol. 14, 880-887
